# Ovarian Cancer Screening: Lessons about Effectiveness

**DOI:** 10.3390/diagnostics8010001

**Published:** 2017-12-29

**Authors:** Edward J. Pavlik

**Affiliations:** Division of Gynecologic Oncology, Department of Obstetrics and Gynecology, University of Kentucky Chandler Medical Center-Markey Cancer Center, Lexington, KY 40536, USA; Epaul1@uky.edu

Ovarian cancer screening has been described in scientific reports [1,2,3,4], as well as in reviews and summaries. Scientific reports contain the facts of a study, while reviews and summaries present interpretations. Presented here are scientific reports which add considerable information to the area of early stage ovarian cancer detection and the application of this detection to ovarian cancer screening. In the present reports:

Froyman and collaborators have assessed and compared the performance of different ultrasound-based International Ovarian Tumor Analysis (IOTA) strategies and subjective assessment for the diagnosis of early stage ovarian malignancy. This important study establishes that the approaches that are taken present a good discrimination between early stage ovarian malignancy and benign abnormalities of the ovary [5].

Baldwin and co-investigators have realized that oophorectomy confers protection against ovarian cancer to the population that has undergone this surgical procedure. As a consequence, risk estimates of ovarian cancer must be adjusted for this protection so that true risk is not underestimated. When these adjustments were made, the rates of ovarian cancer were substantially higher when salpingo-oophorectomy was considered [6].

Ore and associates have examined how frequently and confidently healthy women report symptoms during surveillance for ovarian cancer. They found that the frequency of symptoms relevant to ovarian cancer was more than two hundred times higher than the occurrence of ovarian cancer and that 80.1% of women expressed confidence in the symptoms they reported [7].

Miller and her investigational team compared complications of surgical intervention for participants in the Kentucky Ovarian Cancer Screening Program to results from the Prostate, Lung, Colorectal, and Ovarian Cancer Screening trial (PLCO). They report that complications resulting from surgery performed in the Kentucky Ovarian Cancer Screening Program were infrequent and significantly fewer than reported in the Prostate, Lung, Colorectal and Ovarian Cancer Screening trial. Complications observed were mostly minor (93%) and were more common in cancer versus non-cancer surgery [8].

Ormsby and collaborators present arguments in favor of serial ultrasonography as an alternative to immediate surgery so that any benign abnormality will have the opportunity to resolve. Ultimately, this report presents arguments relative to the benefits of surveillance [9].

Ed Pavlik presents ten critical considerations for ovarian cancer screening, some of which have not been realized in published ovarian screening study reports. These considerations are presented in depth along with illustrations of how they impact the outcomes of ovarian cancer screening trials. These considerations highlight effects that have an important bearing on ovarian screening outcomes and their interpretations [10].

Michael Andrykowski presents considerations that have psychological and behavioral impacts on individuals participating in ovarian screening. His findings suggest that a “normal” screening test result can have psychological benefits, including increased positive affect and beliefs in the efficacy of screening. Moreover, any psychological or behavioral harms attributable to ovarian cancer screening are generally very modest in severity and duration, and might be counterbalanced by psychological benefits accruing to women who participate in routine ovarian cancer screening and receive normal test results [11].

Koshiyama and collaborators present current issues that are related to ovarian cancer and screening. They report that the efficacy of ovarian cancer screening may be higher in Asia than in Europe and the USA. These investigators review the re-analysis of PLCO screening data when cancers presenting more than one year after screening are excluded and show a significant survival benefit in the PLCO screening. They highlight their views by considering the difficulties of detecting Type II ovarian carcinomas [12].

Chris Smith examines the effects that ovarian cancer has on patients and their families. The rigors of treatment conspire with the inevitability of recurrence in the eyes of this first year resident in Obstetrics and Gynecology. He postulates that in the absence of effective therapies, early detection holds the greatest promise [13].

Fred Ueland relates the 50 year history of biomarkers and ultrasound in the context of ovarian cancer. He emphasizes the serial application of both biomarkers and ultrasound. Importantly, he looks to what the future may bring with regard to the utilization of biomarkers and ultrasound in routine patient exams [14].

Taken together, these authors have provided both original data and overviews of ovarian cancer screening studies that enhance the present interpretation of this type of screening.
